# Gas Chromatographic/Mass Spectrometric Analysis of Volatile Metabolites in Bovine Vaginal Fluid and Assessment of Their Bioactivity

**DOI:** 10.1155/2011/256106

**Published:** 2011-10-25

**Authors:** R. Sankar, G. Archunan

**Affiliations:** ^1^Department of Animal Science, Bharathidasan University, Tiruchirappalli 620 024, India; ^2^Department of Animal Behaviour & Physiology, School of Biological Sciences, Madurai Kamaraj University, Madurai 625 021, India

## Abstract

The chemical profiles of vaginal fluid collected from cows in oestrus and nonoestrus were analysed by gas chromatography-mass spectrometry (GC-MS) to establish any qualitative differences that might have potential value in bovine biocommunication. Eight different organic compounds were detected using the two chromatograms. The chemical profiles of oestrus vaginal fluid were distinguished significantly by the presence of three specific substances, namely, trimethylamine, acetic acid, and propionic acid that were not present in nonoestrus phase. The oestrus specific synthetic compounds were rubbed onto the genital region of nonoestrus animals (dummy cows), and the bulls were allowed to sniff the genital region and observed sexual behaviours. The statistical significance was higher (*P* < 0.001) in bulls exhibiting repeated flehmen and mounting behaviours towards the mixture of acetic acid, propionic acid, and trimethylamine as compared to test these compounds separately. It was concluded that the volatile substances present in the bovine vaginal fluid during oestrus may act as chemical communicators.

## 1. Introduction

The role of pheromones in reproduction has been well documented in several species of mammals [1–4]. The pheromonal cues excreted through faeces, urine, vaginal secretions, saliva, and specialised scent glands play a significant role in sexual and social behaviour [5]. In cattle, it is known that bulls can detect pheromone odours and differentiate between oestrus and nonoestrus urine or vaginal fluid [6–8]. The physical and chemical properties of bovine vaginal mucus have been of long standing interest to reproductive biologists because the mucus enhances conception by facilitating viability and transport of sperm [9]. The oestrus female attracts the male and informs its receptive state by means of chemical signals deriving from vaginal discharge [10] and urine [11]. There is considerable evidence that olfactory components produced from vaginal fluid influence the male sexual behaviour in buffaloes [12], hamsters [13], sheep [14], and bovines [15, 16]. In addition, it has reported that vaginal secretions of heifers at oestrus stimulated sexual activity and mounting behaviour [17]. 

The individual animal may produce several volatile compounds from a single source but the influence of pheromone activity may be one compound or a mixture of compounds. Hence, it is necessary to analyse the bioactivity of individual volatile compounds identified in the bovine vaginal fluid. However, identification of the volatile compounds in bovine vaginal fluid and knowledge of how their biological activity may influence sexual behaviour remained to date. The present investigations were designed to analyse the chemistry of vaginal fluid and assess their bioactivity. 

## 2. Materials and Methods

### 2.1. Sample Collection

Vaginal fluid was collected from twelve healthy cows,* Bos taurus* at the Exotic Cattle Breeding Centre, Tanjore, India. They were artificially bred, and the females were approximately 20 to 30 months old, and the males were 30 to 36 months old. The animals were fed a standard diet in which the presence of the chemicals has entirely differed from the identified compounds from the vaginal fluid. The same diet was provided throughout the study. Examination per rectum of each heifer was performed regularly at one- or two-week intervals to verify the normal morphological changes in the internal organ of uterus. Since the detection of vaginal fluid pheromones by bulls is more important for the success of oestrus detection under natural conditions, the samples were collected on the basis of bull behaviours such as licking, sniffing, flehmen, and mounting during oestrus and nonoestrus phases under natural conditions. The technique involved rectal massage of the reproductive tract or an infusion tube placed within the vagina prior to a.i. The samples were screened through cheese cloth or nylon mesh (60–120 *μ*m) at the time of collection. Immediately after screening, the samples were stored frozen at −20°C and analyzed by gas chromatography mass spectrometry (GC-MS).

### 2.2. Sample Analysis

The samples collected from the particular stage as per the experimental protocol were pooled to minimise the effect of individual variation. In a preliminary study, nine organic solvents, namely, n-hexane, acetone, methanol, ethanol, petroleum ether, diethyl ether, chloroform, dichloromethane, and benzene were used to extract the compounds from the vaginal fluid samples. Among the different solvents used, the bull exhibited maximum response (i.e., Flehmen) when exposed to the sample dissolved in diethyl ether. Hence, this solvent was used throughout the study. Triplicate 15 mL of samples were taken from the pooled samples and separately mixed with 15 mL of diethyl ether. The supernatant was filtered through a silica-gel column (60–120 mesh) 30 min at room temperature. The filtered extract was reduced to 1/5 of its original volume by cooling with liquid nitrogen to condense it. 

The sample was fractionated, and chemical compounds were identified by GC-MS (QP-5050, Shimadzu). Two microlitres of extract were injected into the GC-MS system on a 30 m glass capillary column with a film thickness of 0.25 *μ*m (30 × 0.2 mm i.d. coated with UCON HB 2000) using the following temperature program: initial oven temperature of 40°C for 4 min, increasing to 250°C at 15°C/min, and then held at 250°C for 10 min. The GC-MS was run under computer control at 70 eV. The solvent (diethyl ether) peak was seen at 4.0 min. The vaginal fluids were analyzed repeatedly six times and subjected to cross-checking and confirmation. The identified compounds were then compared with the standard run under the same conditions. These data were already stored in a compact library of chemical substance (NIST 6221B).

### 2.3. Behavioural Assay

The synthetic compounds were procured and individually applied manually onto the genital region of nonoestrus animals (dummy cows). The bulls were allowed to sniff the genital region of experimental cows for a period of 30 min. The synthetic compounds were soaked with diethyl ether on cottonwool. The diethyl ether was used for GC-MS analysis as well as dissolving the synthetic compounds in various concentrations for example, 0.5%, 1.0%, 2.0%, 5.0%. Since 1% concentration of oestrus-specific compounds were found to be effective in eliciting the flehmen and other behaviours of penile erection and mounting, the same concentration was taken in the ratio of 1 : 1 : 1 and used throughout the experiment to assess the bioactivity. The duration of Flehmen behaviour exhibited by the bulls in response to dummy cows that received synthetic (oestrus and nonoestrus) compounds was recorded. Subsequently, other behaviour responses like penile erection, mounting, and act of copulation were also recorded.

### 2.4. Statistical Analyses

The data were compiled using SPSS statistical software 10th version and subjected to analysis of variance (ANOVA) with post hoc comparison (one-way) using Duncan's Multiple Range Test (DMRT).

## 3. Results 

**Figure 1:**
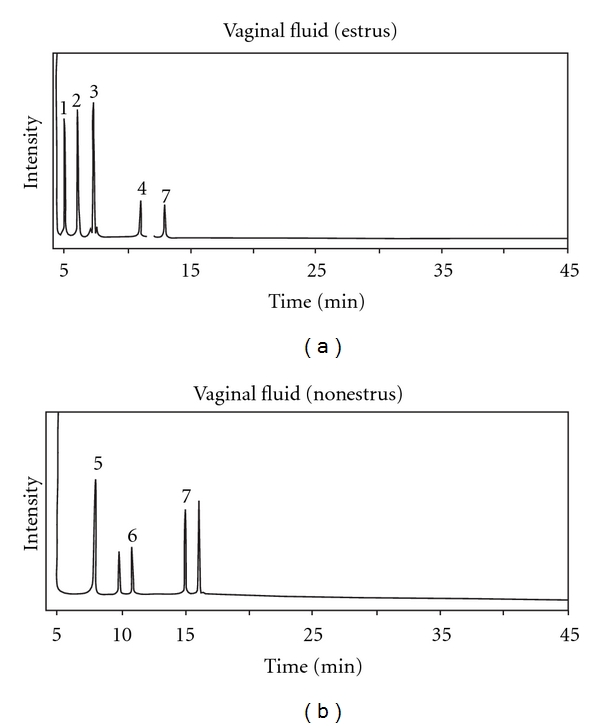
Gas chromatographs of the vaginal fluid compounds identified in oestrus and nonoestrus stages in bovine *Bos taurus*.

**Figure 2:**
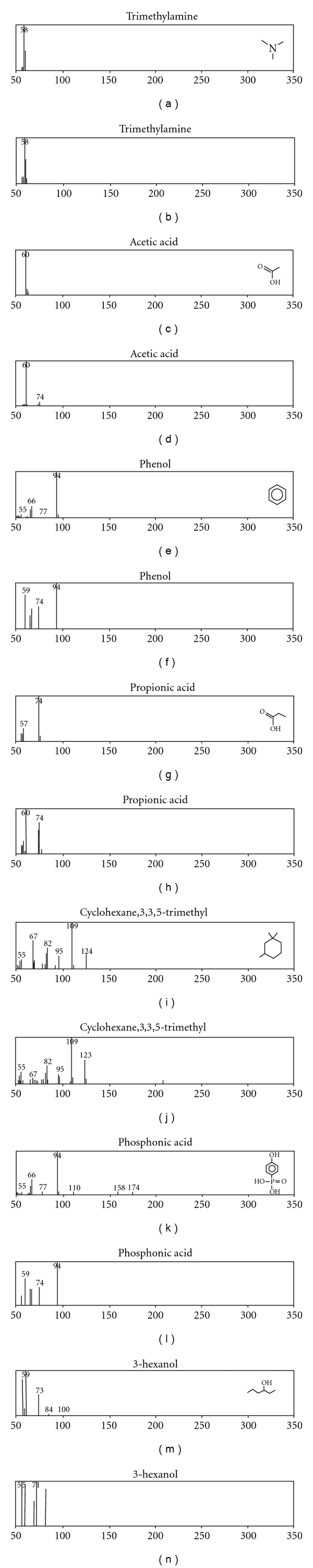
Mass spectrometry of the vaginal fluid compounds identified in the two estrus stages in bovine *Bos taurus. *

 The GC-MS profiles shown in Figures 1 and 2 are the representative of vaginal fluid obtained in the oestrus and nonoestrus periods. The vaginal fluid of oestrus showed five peaks and of nonoestrus exhibited three peaks. Eight different peaks were noted in the vaginal fluid of two different phases (Figure 1). A single volatile, that is, 3-hexanol, was found in both the phases whereas other two volatiles, cyclohexane 3,3,5 trimethyl and phosphonic acid, were found only in nonoestrus vaginal fluid (Table 1). Of these, trimethylamine, acetic acid, and propionic acid were unique in the oestrus phase but were absent in the nonoestrus. The peaks between 5 and 6 and after the peak 7 in nonoestrus are referred as contaminants and not matching with any related compounds. The volatile compounds identified in the vaginal fluid have the molecular weight range between 59 and 174 dalton (Table 1). 

The statistical analyses showed that the flehmen behaviour of bulls was greatly influenced by the mixture of three synthetic compounds (*P* < 0.001) than that of individual compounds and control sample (Table 3). The number of mounting activity and flehmen (Table 3) was higher in response to mixture of the three compounds than that of individual and in combinations. 

The results of Table 2 summarises the flehmen and mounting behaviour performed by the male responder on exposure to synthetic compounds. Among the individual synthetic compounds tested the combination of acetic and propionic acid showed higher sexual behaviour than that of individual and combination of acetic acid and trimethylamine; propionic acid and trimethylamine. 

## 4. Discussion 

The present results revealed that the acetic acid, propionic acid, and trimethylamine appeared during oestrus phase but were not found in the nonoestrus phase. Among the compounds identified in oestrus vaginal fluid, the acetic and propionic acids belong to fatty acids, and the trimethylamine is in amine group. The identification of volatile fatty acids in the bovine vaginal fluid is consistent with the report of the predominant presence of short chain aliphatic acids, acetic acid, prop-, isotonic, in vaginal secretion of rhesus monkey [18]. Such volatile aliphatics have also been demonstrated in the vaginal secretion of an inside range of primates, including human female [19]. Furthermore, [20] reported that acetic, propionic, and isobutyric acid can act as pheromone in chimpanzee.

 In the present study the bulls exhibited high frequency of flehmen response when exposed to oestrus sample as well as mixture of synthetic compounds tested. It clearly indicates that these three oestrus-specific compounds probably act as sex attractants (Table 1). It has been demonstrated that the olfactory chemical signals produced from oestrus females are mediated through the vomeronasal organ (VNO) facilitating the matting behaviour through expression of flehmen response in many species [20, 21]. The present findings further confirm that the identified compounds in oestrus and to activate the bull to mount are the sex attractants. 

It is reported that in some mammals, pheromones are not single compounds but a mixture [22]. Reference [23] reported that three oestrus-specific compounds, namely, acetic acid, propionic acid, and 1-iodo undecane of cattle (*Bos taurus*) were involved in the attraction of the opposite sex. Oestrus-specific bovine urinary signal, 1-iodo undecane, is identified successfully in our laboratory by gas chromatography linked with mass spectrometry [7]. Similarly, in the present study the mixture of acetic, propionic, and trimethylamine revealed high incidence of precopulatory behaviour. The oestrus indication may have been initiated through urine followed by vaginal secretion, and accordingly the bull responded. It is, therefore, possible that the chemical signals produced from urine and vaginal fluid may act together for initiation of the precopulatory behaviours and successful coitus. 

## 5. Conclusion

Chemical signals are less likely to be based on the presence of unique chemical compounds than on the relative ratios of concentrations in a complex mixture [24]. This concept is commonly termed as “Bouquet effect” [25, 26], also proposed that volatile compounds in specific ratios and in the presence of unique components might confer pheromonal effects. Consistent with this suggestion, the present study also reveals that estrus vaginal fluid contains three specific compounds, which may be the basis of estrus odours. It is noteworthy that the present study on behaviour responses showed similar results using the mixture of synthetic compounds to that of oestrus vaginal fluid. We conclude that the specific volatile fatty acids present in bovine vaginal fluid during oestrus appear to be involved in sexual communication.

## Figures and Tables

**Table 1:** Volatile compounds identified in bovines' vaginal fluids during the oestrous cycle.

Peaks	Molecular weight	Identified compounds	Oestrus	Nonoestrus
1	59	Trimethylamine	+	−
2	60	Acetic acid	+	−
3	94	Phenol	+	−
4	130	Propionic acid	+	−
5	124	Cyclohexane,3,3,5-Trimethyl	−	+
6	174	Phosphonic acid	−	+
7	102	3-hexanol	+	+

+ Indicates the presence of the compound; − Indicates the absence of the compound.

**Table 2:** Bioactivity of oestrus-specific compounds.

S. no.	Oestrus-specific compounds	Duration of flehmen (seconds)	Number of mounts
1	Acetic acid	5.52 ± 0.02	8.40 ± 0.46
2	Propionic acid	5.58 ± 0.22	8.80 ± 0.52
3	Trimethylamine	4.20 ± 0.08	5.85 ± 0.52
4	Phenol	2.63 ± 0.02	3.96 ± 0.40
5	Mixture of acetic acid and propionic acid	6.96 ± 0.52	8.84 ± 0.52
6	Mixture of acetic acid and trimethylamine	4.68 ± 0.54	6.08 ± 0.40
7	Mixture of propionic acid and trimethylamine	5.13 ± 0.33	5.60 ± 0.06
8	Mixture of acetic acid, propionic acid and trimethylamine	8.60 ± 0.01	10.94 ± 0.87
9	Phosphonic acid (negative control; non-oestrus sample)	2.19 ± 0.33	3.63 ± 0.36

Values are mean ± S.E of six observations.

**Table:** (a)

ANOVA						
		Sum of squares	df	Mean squares	*F*	*P*

VAR 00001 *Duration of flehmen *	Between squares	189.995	8	23.749	76.874	0.000**
	Within groups	13.902	45	.309		
	Total	203.897	53			

VAR 00003 *Mounting behaviour *	Between squares	294.012	8	36.752	134.779	0.000**
	Within groups	12.271	45	.273		
	Total	306.283	53			

**Level of significance at (*P* < 0.001).

**Table:** (b)

VAR 00001 DUNCAN							

	Subset for alpha = .05						
VAR 00002	*N* = 6	1	2	3	4	5	6

Phosphonic acid	6	2.1917					
Phenol	6	2.6333					
Trimethylamine	6		4.2083				
Acetic + TMA	6		4.6817	4.6817			
Propionic + TMA	6			5.1367	5.1367		
Acetic acid	6				5.5250		
Propionic acid	6				5.5800		
Acetic + propionic	6					6.9600	
*Acetic + propionic + TMA*	6						*8.6067*
Significant		0.176	0.147	0.163	0.199	1.000	1.000*

*Means for groups in homogenous subsets are displayed comparison of means using DMRT*. The DMRT test showed that the mixture of acetic acid, propionic acid, and trimethylamine was found to be significant (*P* < 0.001) compared to those of acetic acid, propionic acid, Trimethylamine, phosphonic acid, and phenol separately as well as combinations.

**Table:** (c)

VAR 00001 DUNCAN					

	Subset for alpha = .05				
VAR 00002	*N* = 6	1	2	3	4

Phosphonic acid	6	3.6350			
Phenol	6	3.9683			
Propionic + TMA	6		5.6067		
Trimethylamine	6		5.8567		
Acetic + TMA	6		6.0817		
Acetic acid	6			8.4033	
Acetic + propionic	6			8.8467	
Propionic acid	6			8.8867	
*Acetic + propionic + TMA *	6				*10.9400*
Significant		0.275	0.144	0.137	1.000*

*Means for groups in homogenous subsets are displayed comparison of means using DMRT.* The DMRT test showed that the mixture of acetic acid, propionic acid, and trimethylamine was found to be significant (*P* < 0.001) compared to those of acetic acid, propionic acid, trimethylamine, phosphonic acid, and phenol separately as well as combinations. Abbreviation: TMA: trimethylamine.
